# Melatonin Postharvest Treatment in Leafy ‘Fino’ Lemon Maintains Quality and Bioactive Compounds

**DOI:** 10.3390/foods12152979

**Published:** 2023-08-07

**Authors:** Fátima Badiche-El Hilali, Juan Miguel Valverde, María E. García-Pastor, María Serrano, Salvador Castillo, Daniel Valero

**Affiliations:** 1 Department of AgroFood Technology, EPSO-CIAGRO, University Miguel Hernández, Ctra. Beniel km. 3.2, 03312 Orihuela, Alicante, Spain; fbadiche@umh.es (F.B.-E.H.); jm.valverde@umh.es (J.M.V.); m.garciap@umh.es (M.E.G.-P.); scastillo@umh.es (S.C.); 2 Department of Applied Biology, EPSO-CIAGRO, University Miguel Hernández, Ctra. Beniel km. 3.2, 03312 Orihuela, Alicante, Spain; m.serrano@umh.es

**Keywords:** bioactive compounds, firmness, melatonin, organic, postharvest, quality

## Abstract

Spain is a great producer of organic lemon; however, it is necessary to reduce the losses caused by post-harvest diseases. Melatonin (MEL) is a naturally occurring compound with physiological functions in fruit growth and ripening and is able to modulate postharvest ripening and senescence, most of it being concentrated in climacteric fruit. Thus, the aim of this study was to apply MEL to organic lemon fruit with stems and leaves (LEAF) and to organic lemon without those components (LEAFLESS) after harvesting and storage during 21 days at 2 °C to understand the effects of this treatment on the fruit quality. For this purpose, two experiments were carried out. First, MEL was applied at 0.01 mM, 0.1 mM and 1.0 mM by immersion for 15 min on lemon fruits, and the quality parameters and bioactive compounds of the fruit were analysed. Subsequently, a second experiment was carried out where the best concentration (1 mM) was selected and another time (15 and 30 min) was added, with the same quality parameters being analysed. As a result, we observed that all MEL treatments showed positive effects on weight loss reduction, softening (higher fruit firmness), total acidity and lower colour changes. Total phenols increased in MEL-treated lemons, both in peel and juice. For the three concentrations tested, the best efficiency was obtained with MEL at 1.0 mM, while LEAF lemons were the most effective. In conclusion, lemons containing stems and leaves (LEAF) improved preservability by using MEL at 1.0 mM with better organoleptic quality and enhanced phenolic compounds.

## 1. Introduction

Nowadays, in the marketing of some citrus fruits, the option of selling the fruit together with stem and leaves is possible, mainly for clementines and oranges. The presence of the leaves with the fruit means that the product is more natural and fresher [1]. There is not enough knowledge about the role of the leaf in maintaining quality during post-harvest. However, some studies have been carried out on pineapple and have concluded that the crown plays an important role in post-harvest quality maintenance. The fruit’s detachment from the pineapple crown after harvest increases internal browning, reactive oxygen species (ROS), cell membrane damage, biosynthesis and oxidation of compounds [2]. These events directly influence the quality of the fruit.

Size, shape, skin thickness, visual appearance or colour as well as the percentage of juice, sugars, acidity or bioactive compounds are quality parameters that determine consumer acceptability [3]. One of the major postharvest disorders that affects lemon fruit quality is decay incidence, which is mainly caused by *Penicillium digitatum* and causes huge economic losses for the lemon industry worldwide [4]. Currently, postharvest diseases have been controlled mainly by the application of conventional fungicides such as imazalil (IZ), sodium ortho-phenyl phenate (SOPP), thiabendazole (TBZ) or different mixtures of these compounds [5]. The use of fungicides is prohibited in organic crops. Several non-thermal technologies have also emerged in recent decades for the treatment of food crops, such as UV-C irradiation or electrical pulses, although their potential to negatively influence sensory attributes has been highlighted [6]. Therefore, compared to conventional lemons, organic lemons present additional problems until they reach the final consumer in optimum quality conditions [7]. They are more susceptible to postharvest diseases, so in order to consolidate a more sustainable agriculture without adverse effects on consumer health, new alternatives have emerged in food production, such as the use of elicitors.

Melatonin (MEL) or N-acetyl-5-methoxytryptamine is an indoleamine that was first discovered from the pineal gland of vertebrates [8] and, five decades later (1995), in the plant kingdom. MEL, as a phytohormone, is involved in a wide range of physiological processes in plants, from germination to ripening and senescence as well as amelioration of several abiotic stresses [9,10,11]. The biosynthetic pathway of MEL starts with the amino acid tryptophan, which is converted into serotonin and via N-acetylserotonin produces MEL and is regulated by six enzymes that are essential for maintaining the endogenous levels [12]. In fruits, the endogenous concentration of MEL changes during development and maturation, reaching a peak after fruit setting and progressively with the onset of ripening, as has been observed in sweet cherries [13]. Based on this statement, preharvest applications of MEL were effective on increasing yield, the content of bioactive compounds (polyphenols and anthocyanins), as has been reported for pomegranate [14,15], and enhancing the quality attributes of sweet cherries such as colour, firmness, total soluble solids (TSS) and titratable acidity (TA) [16]. As a postharvest treatment, MEL could be useful to maintain and improve the postharvest quality traits in fruit and to reduce the spoilage and decay, although different effects have been obtained depending on the fruit species [17]. In addition, MEL was also effective on increasing the content of bioactive compounds and the antioxidant activity of several fruit commodities [18] but also alleviating several abiotic stresses, the most studied being chilling injury [19].

However, the use of MEL during postharvest storability of citrus fruits has been poorly studied, and few data have been reported. In ‘Newhall’ navel oranges, MEL induced a reduction in weight loss and respiration rate and increased the organoleptic quality traits [20]. The immersion with MEL (0, 10, 100 and 1000 nmol) of ‘Washington’ navel oranges and storage at chilling temperature was effective in reducing the CCI index (the standard parameter used in the citrus industry to determine the ripening stage of citrus fruit by colour [21]) and water loss and preserved the skin colour, the best results being obtained for MEL at 1000 nmol [22]. The citrus industry is providing new formats of several citrus fruit with the presence of stalks and leaves; for that reason, the demand for this new citrus presentation by consumers is growing. However, lemons are normally harvested without stems and leaves and are susceptible to quality losses during post-harvest storage. Therefore, the aim of this work was to evaluate the role of melatonin as an elicitor agent in the behaviour of lemon fruits, harvested both with stems and leaves (LEAF) and without them (NO LEAF), in order to know if it can preserve or improve the quality of the fruit during postharvest storage.

## 2. Materials and Methods

### 2.1. Plant Material and Experimental Design

**Figure 1:**
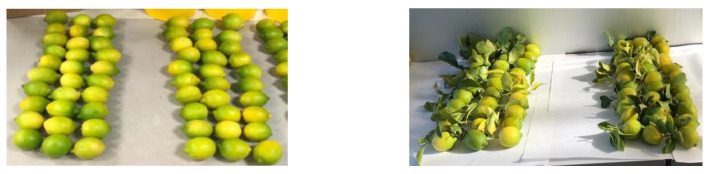
Photography of ‘Fino’ lemons with stalks and leaves (**right**, LEAF) and without (**left**, NO LEAF) used in the experiments.

The experiments were carried out with organic lemon trees that were 20 years old and grafted on *Citrus macrophylla* rootstock (*Citrus limon* (L.) Burm. f) ‘Fino-95’, growing in a commercial orchard located in La Matanza (Alicante, Spain). The crop was located in an area with a Mediterranean climate, characterised by an average annual temperature of 18.2 °C and a rainfall of 283 mm. The lemon trees were cultivated in accordance with the current organic farming regulations, using a planting pattern of 6 × 5 m and during the growth cycle of 2021–2022. Two experiments were carried out, Harvest 1 and Harvest 2. For experiment 1, or Harvest 1, 30 lemon trees in good vegetative condition were used, and 10 fruits were picked at random from each tree: 150 of them were cut with a part of the stalk and a couple of leaves (LEAF) and the other 150 were cut by the stalk (NO LEAF) according to the standard procedure for commercialisation, as shown in Figure 1.

At the laboratory, 8 lots of 30 fruits were selected (240 fruits) in the same day, from the same sub-region and the same producer, of which 4 lots were with leaves and 4 were without leaves. Both types of lemons were treated with melatonin at 0.01 mM, 0.1 mM and 1.0 mM concentrations by dipping for 15 min (based on previous experiments [16]), while control fruits were immersed in distilled water. After treatments, the fruits stood on the bench for air drying before being stored under cold conditions at 2 °C and an Relative Humidity (RH) of 90%. Analytical determinations were performed after 21 days plus 2 days at 20 °C (shelf-life), and, additionally, 30 fruits with leaves and 30 without leaves were used for day 0 determinations. For the second experiment, or Harvest 2, the conditions were the same; we used new lemons with the same features. The only difference between the harvests was the number of lemons used, which was reduced from 8 lots to 4 lots, 2 lots of 30 lemons with leaves and 2 lots of 30 lemons without leaves, since only one concentration of melatonin (1 mM) was used because it was the one with the best results. In addition, two different times for dipping (15 and 30 min) were performed.

### 2.2. Lemon Fruit Quality Characteristics

Quality parameters (physiological weight loss, fruit firmness, respiration rate, colour, total soluble solids content, titratable acidity and electrolyte leakage) were individually measured for each lemon fruit (data are the mean ± SE, *n* = 30). Physiological weight loss was determined by weight of the recently harvested fruit and that obtained at each sampling date and expressed in percentage (data are the mean ± SE, *n* = 30). For fruit firmness, a texturometer (TA-XT2i Texture Analyzer, Stable Microsystems, Godalming, Surrey,, UK) was used with a flat probe of 10 cm. The percentage of deformation was 3% of the fruit diameter, and results were expressed as N mm^−1^ (Data are the mean ± SE, *n* = 30). The respiration rate was analysed at room temperature; each lemon fruit was placed in a 0.5 L glass jar for 60 min. Then, 1 mL of the atmosphere generated in the headspace was sampled, and the CO_2_ content was quantified using a gas chromatograph (Shimadzu: Kyoto, Japan) coupled with a thermal conductivity detector. The results were expressed in mg CO_2_ kg^−1^ h^−1^. Colour was measured at three points along the equatorial fruit perimeter by using a Minolta colorimeter (CRC400, Minolta, Osaka, Japan), and the results were expressed using the Citrus Colour Index (CCI), which was determined by measurements of the basic parameters of L*, a* and b* using a colorimeter. C*, Ho and CCI were calculated using the formulas as follows: C* = (a*2 + b*2)1/2, Ho = arctan (b*/a*) and CCI = 1000 a*/(L*·b*), respectively. The content of TSS was quantified in each juice sample using a digital refractometer (Hanna Instruments, Woonsocket, RI, USA). Moreover, TA was measured in the same juice sample by the automatic titration (785 DMP Titrino, Metrohm, Herisau, Switzerland) of 0.5 mL juice neutralised with different volumes of 0.1 N NaOH until a pH of around 8.1 was achieved. Electrolyte leakage (EL) was evaluated in the peel tissue, following the method described by McCollum and McDonald [23] with some modifications. First, slices of the three replicates per treatment were cut to 4 mm thickness in the equatorial zone of the lemon fruit. Fifteen discs were extracted for each replicate using a 0.5 cm diameter cork borer. After 3 rinses of 3 min for each replicate with deionized water, they were subjected to constant shaking with 50 mL of deionized water at room temperature. After 30 min, the initial electrical conductivity (C1) was measured using a Crison conductivity meter. The samples were frozen and then brought to 121 °C for 15 min. Total conductivity (C2) was evaluated with samples at room temperature (20 °C). EL was calculated as (C1/C2) × 100.

### 2.3. Extraction and Quantification of Total Phenolic Content

The Folin-Ciocalteu method was used as previously described by García-Pastor et al. in 2020 [24]. A stainless-steel lemon peeler was used to obtain the flavedo tissue by homogeneously splitting several 0.5 mm thick slices of lemon peel. For lemon juice extraction, a domestic squeezer (‘Citromatic’, Braun Española S.A., Barcelona, Spain) was used for the preparation of the juices by carefully squeezing the fruits by hand to avoid contamination by the components in albedo. Thus, 2 g of flavedo or 2 mL of juice were added to a solution of water:methanol (2:8) and 2 mM NaF (to inhibited the degradation of the phenolics by the enzyme polyphenoloxidase) and a homogeniser with an Ultraturrax homogeniser (T18 basic, IKA, Berlin, Germany) at maximum speed for 1 min. The extract was centrifuged (at 4 °C and 10,000× *g* for 10 min). An aliquot (200 μL) was mixed with the Folin reagent and the absorbance was quantified at 760 nm in a UV-Vis spectrophotometer (UV-1900i, Shimadzu, Duisburg, Germany).

### 2.4. Statistical Analysis

The software IBM SPSS Statistics 22.0 (IBM Corp., Armong, NY, USA) was used for statistical analyses. Data are the mean ± SE for each replicate (*n* = 3). The data of the analytical determinations were submitted to two-way analysis of variance with treatment, storage time and type of leaf (Leaf vs. No Leaf) as factors. Means were compared using a multiple range test (Tukey’s test) to examine if differences among treatments, storage time and type of leaf were significant, and the level of significance was established at *p-*value < 0.05.

## 3. Results and Discussion

### 3.1. Effect of Melatonin on Respiration Rate and Weight Loss

**Figure 2:**
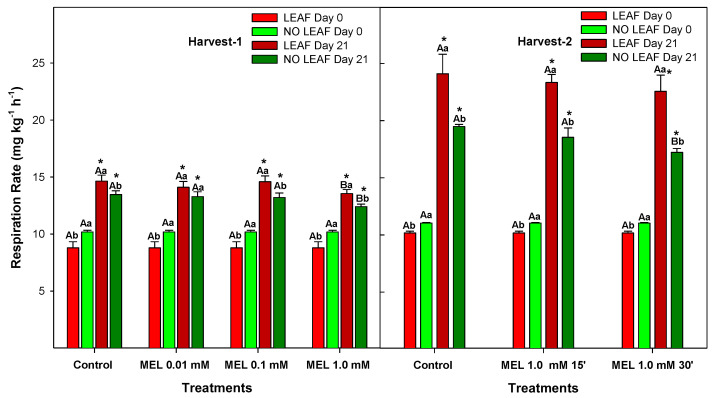
Respiration rates of ‘Fino’ lemons with LEAF and NO LEAF as affected by MEL treatments at 0.01, 0.1 and 1.0 mM (Harvest 1) and MEL at 1.0 mM during 15 and 30 min (Harvest 2) after 21 days of storage. Data are the mean ± SE. Bars with different capital letters denote significant differences between control and MEL treatments, while bars with different small letters denote significant differences between lemons with LEAF and NO LEAF. The asterisk symbol denotes significant differences between both storage days (0 and 21 days) for each type of leaf and treatment.

Respiration consists of a series of enzyme-catalysed reactions, the rate of which is related to temperature [25]. Furthermore, it is a complex of several oxidative reactions due to the breakdown of carbohydrates, mainly glucose, to produce the necessary energy during the storage of fruits in which H_2_O and CO_2_ are produced [26]. Citrus fruits are non-climacteric with slow respiration rates, although respiration of citrus fruits is affected by several factors, including temperature, humidity, gas composition of the atmosphere and handling practices, among others [27]. The respiration rate of ‘Fino’ lemon fruit gradually increased during the 21 days of storage significantly *(p*-value < 0.05), for both Harvest 1 and Harvest 2 and with LEAF and NO LEAF (Figure 2). At 21 days + 2 days of shelf life, control leafless lemons showed a significant lower respiration rate of 13.46 ± 0.34 mg kg^−1^ h^−1^ compared to 10.17 ± 0.15 mg kg^−1^ h^−1^ on day 0 (*p*-value < 0.05). Melatonin-treated NO LEAF lemons tended to have a lower respiration rate than LEAF lemons, with the exception of those treated with 0.01 mM. This result suggests that the lowest dose of 0.01 mM has no effect on this physiological parameter. The respiration rate of fruits with leaves also increased compared to the day of harvest (Day 0). After storage, control LEAF lemons showed a respiration rate of 14.65 ± 0.30 mg kg^−1^ h^−1^, which was about 1% higher than NO LEAF lemons. Leafy lemons treated with melatonin increased their respiration rate, with the exception of those treated with 1 mM that had significantly (*p*-value < 0.05) lower results than the control with 13.55 ± 0.15 mg kg^−1^ h^−1^. In addition, lemons from Harvest 2 showed greater respiration rates than those from Harvest 1.

The respiration rate was significantly higher (*p*-value < 0.05) in lemons with LEAF than it was in those with NO LEAF for both experiments apart from the case of MEL at 0.01 mM. This result was expected since the respiration rate is the sum of both leaves and fruits. It has been reported that the leaves exhibited a significantly higher respiratory intensity (20 fold times) than the fruit [28]. However, the application of MEL at 0.01, 0.1 and 1.0 mM during 15 min (Harvest 1) showed that 1.0 mM was most effective in reducing the respiration rate in both LEAF and NO LEAF lemons. Considering this result, in the second experiment (Harvest 2), 2 dipping times (15 and 30 min) and the MEL concentration of 1 mM were assayed. The results were similar, but the dipping time of 30 min led to a significantly lower respiration rate than that obtained for 15 min. A high respiration rate during storage leads to excessive consumption of nutrients such as sugars, organic acids and amino acids, thus affecting the lemon quality, as occurred in control lemons. The application of MEL at 1.0 mM could retard lemon quality deterioration and delay the senescence process by reducing the respiration intensity, as has been observed for navel oranges [20], pears [29] and sweet cherries [30]. In addition, the oranges study concluded that melatonin significantly delays physiological senescence through a reduction of post-harvest respiration. Similar results were also found in [31]. However, the mechanism of action on inhibiting the respiration rate during postharvest storage of fruits, as well as the molecular and biochemical regulation, deserves further investigation.

**Figure 3:**
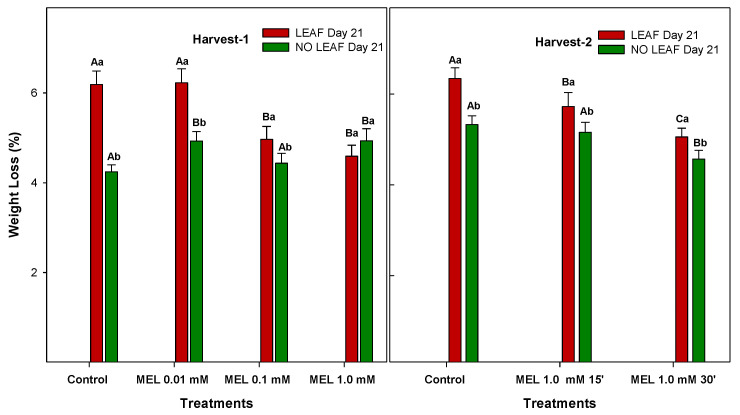
Percentage of weight loss of ‘Fino’ lemons with LEAF and NO LEAF as affected by MEL treatments at 0.01, 0.1 and 1.0 mM (Harvest 1) and MEL at 1.0 mM during 15 and 30 min (Harvest 2) after 21 days of storage. Data are the mean ± SE. Bars with different capital letters denote significant differences between control and MEL treatments, while bars with different small letter denote significant differences between lemons with LEAF and NO LEAF.

The weight loss of fruits and vegetables during storage is mainly due to the decrease of water during transpiration, which is one of the main causes of the damages of lemons that results in quality losses. Once the lemon has been harvested, transpiration processes continue, where water in a vapour state goes through the stoma and the epidermis, causing the loss of fruit weight [25]. Then, physiological water loss is considered one of the most important factors related to fruit quality and various postharvest disorders. There are many factors affecting water loss, which are classified into 3 categories: pre-harvest, harvest and postharvest factors. At postharvest, the most important are cold temperature and relative humidity of the chamber [32]. During 21 days of storage, and regardless of treatment, the weight loss of ‘Fino’ lemons exhibited a progressive enhancement, the increase being significantly higher (*p*-value < 0.05) in LEAF than NO LEAF fruits in both harvests (Figure 3). In LEAF lemons from Harvest 1, both control and MEL at 0.01 mM reached the highest weight loss (≈6%), while the lowest was obtained for those fruits treated with MEL at 1.0 mM. In lemons from Harvest 2, MEL-treated fruits at 1.0 mM and 30 min showed the lowest weight losses (≈4.5%), with a similar behaviour for NO LEAF lemons. However, at Harvest 1, NO LEAF lemons show higher weight losses than the control (*p*-value < 0.05), with the exception of 0.1 mM melatonin.

It has been reported that water loss over 5% of the initial fruit weight will affect the visual appearance, with major symptoms being wilting, peel desiccation, membrane disruption and onset of the senescence, which will limit fruit marketability and cause economic losses [32,33]. Comparing the two preservation methods (LEAF, NO LEAF), it has been observed that lemons without leaves showed lower weight losses than when the fruit kept the leaves. This could be due to the larger surface of the leaf promoting greater transpiration [34]. The results show that melatonin treatment does not affect lemons without leaves; however, it does have a beneficial effect on lemons with leaves. In the orange study [20], the researchers concluded that MEL treatment caused a significant decrease in weight loss compared to the control. Similar results have also been found in mandarins and strawberries as well as in sweet cherries and other ‘Kinnow’ fruits and vegetables [30,31,35,36]. Physiological postharvest weight loss is usually attributed to both water loss (by transpiration) and respiration. In this report, since all the fruits were in the same cold rooms with identical temperature and relative humidity, the MEL treatment on reducing the weight loss could be attributed to the reduced respiration rate, as has been observed in navel oranges [20].

### 3.2. Effect of Melatonin on Lemon Quality Characteristics

The quality of fruits refers to a series of characteristics that determine their degree of acceptance by the consumer, and in the case of lemon colour, firmness, electrolyte leakage, total soluble solids and titratable acidity are the most important and are related to organoleptic quality.

**Figure 4:**
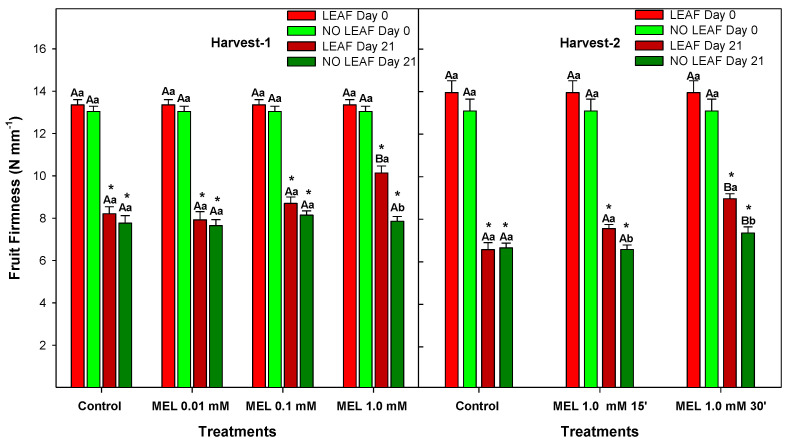
Firmness of ‘Fino’ lemons with LEAF and NO LEAF as affected by MEL treatments at 0.01, 0.1 and 1.0 mM (Harvest 1) and MEL at 1.0 mM during 15 and 30 min (Harvest 2) after 21 days of storage. Data are the mean ± SE. Bars with different capital letters denote significant differences between control and MEL treatments, while bars with different small letters denote significant differences between lemons with LEAF and NO LEAF. The asterisk symbol denotes significant differences between both storage days (0 and 21 days) for each type of leaf and treatment.

Softening is one of the main causes affecting quality deterioration and reduced shelf life of fruits, both climacteric and non-climacteric. Results about firmness are shown in Figure 4. After 21 days of storage, firmness decreased in both control and MEL-treated lemons and also in LEAF and NO LEAF lemons (*p*-value < 0.05) in Harvest 1 and Harvest 2. However, those fruits treated with MEL at 1 mM showed higher firmness compared with the control, with MEL at 0.01 and 0.1 mM (*p*-value < 0.05) being the most effective dose related to this parameter. In Harvest 2, the MEL treatment in LEAF fruits presented higher firmness with respect to the control (*p*-value < 0.05) for both 15 and 30 min. In NO LEAF fruits, firmness was greater in treatment with MEL 1 mM 30’ than in the control and MEL 1 mM 15’ (*p*-value < 0.05).

Comparing LEAF and NO LEAF lemons, there were not significant differences (*p-*value > 0.05), with the exception of MEL at 1 mM, since LEAF lemons showed significantly higher firmness (≈11 N mm^−1^) than NO LEAF (≈7 N mm^−1^) in Harvest 1, with the same behaviour being obtained in Harvest 2 when 15 and 30 min were used.

Firmness of fruit and vegetables is modified throughout post-harvest storage. These changes in texture are related to the turgidity of tissues and, therefore, to the hydrolytic activity of enzymes that are responsible for degrading pectins, celluloses and hemicelluloses of the cell wall [37]. The maintenance of firmness as a consequence of postharvest MEL treatments is a major factor that contributes to preserving fruit quality during storage. Accordingly, MEL at 0.5 mM by dipping 1 h delayed the postharvest ripening of mangoes [33], as well as for bananas, in a concentration-dependent manner in the range of 0.05–0.5 mM [38]. In pomegranates, preharvest application of MEL at 0.1, 0.3 and 0.5 mM was effective in maintaining firmness during 60 days of storage [15]. Thus, results demonstrate that MEL applications, especially at 1.0 mM, retarded the decrease of firmness, which could be related to a delay in cell wall degradation [29].

The organoleptic properties that are essential related to consumer appreciation of lemons are aroma, taste and colour. However, the senescence process during postharvest is manifested by the deterioration of sensory quality attributes, with changes in the levels of sugars, organic acids, and colour deterioration. The total soluble solids (TSS) and titratable acidity (TA) are key parameters for evaluating the quality of lemon juice. They are used as a maturity index in quality measurements [39]. With respect to total soluble solids in our samples, no significant differences (*p*-value > 0.05) were shown between the control and any MEL concentration, neither LEAF and NO LEAF lemons nor between day 0 and day 21 at Harvest 1. At Harvest 2, apart from LEAF and NO LEAF lemons showing significant differences at MEL 1 mM 15 and 30 min (*p*-value < 0.05), we observed the same behaviour (Figure S1). In general, there was not an increase in sugar content or a decrease in acidity of non-climacteric fruits throughout the storage, which is consistent with the progress of the ripening process. The application of MEL on titratable acidity (Figure S2) showed that a reduction (*p*-value > 0.05) was produced in control and MEL-treated lemons after 21 days of storage, being higher in LEAF than in NO LEAF fruits at Harvest 2.

With regard to our results, the application of postharvest melatonin in nectarine fruit by 30 min of immersion showed no significant difference in TSS content between untreated and melatonin-treated fruit after 30 d of storage [35]. However, after 40 days of storage, nectarines showed a decrease in TSS at the highest applied melatonin concentration (1000 µmol L 1), which is consistent with other studies in plums and mangoes [33,40]. Regarding the TA content, several studies show similar results; the application of MEL in nectarines, mangoes and plums showed that there were no differences between the control and treatments. The role of MEL in maintenance acidity has been already reported in other fruits such as navel oranges [20], pears [29] and sweet cherries [30].

**Figure 5:**
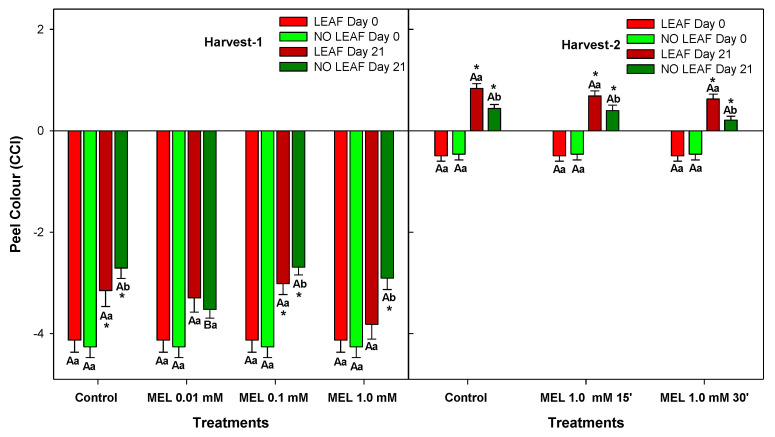
Peel colour of ‘Fino’ lemons with LEAF and NO LEAF as affected by MEL treatments at 0.01, 0.1 and 1.0 mM (Harvest 1) and MEL at 1.0 mM during 15 and 30 min (Harvest 2) after 21 days of storage. Data are the mean ± SE. Bars with different capital letters denote significant differences between control and MEL treatments, while bars with different small letters denote significant differences between lemons with LEAF and NO LEAF. The asterisk symbol denotes significant differences between both storage days (0 and 21 days) for each type of leaf and treatment.

We measured colour (expressed as the Citrus Colour Index, CCI) in the peel of lemons. With respect to peel colour (Figure 5), CCI at Harvest 1 was −4, showing that the skin had a light yellow colour. During storage, CCI decreased in both control and MEL-treated lemons, although LEAF fruits treated with MEL at 1.0 mM did not significantly (*p*-value > 0.05) change with respect to values at harvest (Harvest 1), contrary to NO LEAF lemons since they had the lowest CCI (≈−3). However, in Harvest 2, the lowest CCI (*p*-value < 0.05) was obtained in NO LEAF lemons, independently of duration of treatment (15 or 30 min).

The peel colour is usually associated with the internal quality (flavour and texture), which can affect the purchase decision by the consumers [41]. It is well known that the change in colour from green to yellow in lemon fruits is due to some modifications in the composition and concentration of chlorophylls and carotenoids. According to several studies, melatonin significantly decreases chlorophyll degradation and increases the biosynthesis of carotenoids (including α, β-carotene and lycopene) at transcriptomic and metabolic levels in broccoli, tomatoes and cabbage [42,43,44]. In our work, MEL treatment enhanced colour changes of lemon fruit, as indicated by the higher values of CCI, consistent with navel oranges [20] treated with MEL at 200 μM. In lemons (yellow-coloured citrus), contrarily to oranges, the changes in carotenoids during fruit growth and ripening have been less investigated [45], which might be due to greater activity of phytoene synthase than of phytoene desaturase [46]. In another study of melatonin post-harvest treatment in oranges, something similar occurred, and the color of the control fruit exhibited a strong increase in storage. However, the oranges treated with melatonin at a concentration similar to ours accelerated and significantly improved this change of color [20]. It also occurs in strawberries and sweet cherries [30,31].

**Figure 6:**
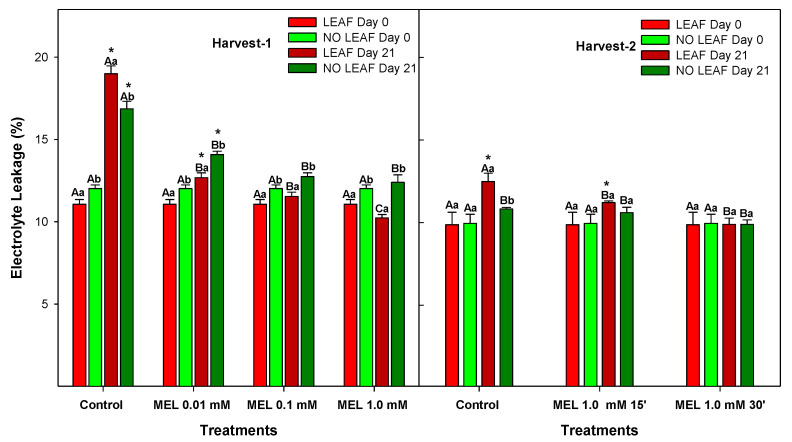
Electrolyte leakage (EL) of ‘Fino’ lemons with LEAF and NO LEAF as affected by MEL treatments at 0.01, 0.1 and 1.0 mM (Harvest 1) and MEL at 1.0 mM during 15 and 30 min (Harvest 2) after 21 days of storage. Data are the mean ± SE. Bars with different capital letters denote significant differences between control and MEL treatments, while bars with different small letters denote significant differences between lemons with LEAF and NO LEAF. The asterisk symbol denotes significant differences between both storage days (0 and 21 days) for each type of leaf and treatment.

The incidence of CI symptoms results from primary and secondary responses to cold temperatures. Changes in the membrane structures and permeability are primary responses. On the other hand, secondary responses consist of ion leakage, a decrease in cellular energy and oxidative damage [47]. Electrolyte leakage (EL) is among the first defence activation events in plants, and membrane permeability can be evaluated by measuring this parameter. Figure 6 shows that EL significantly increased (*p*-value < 0.05) from the beginning to the end of storage in both harvests for controls and Mel 0.01 mM. However, those fruits treated with MEL at 0.1 and 1 mM did not show significant differences during storage. LEAF lemons EL showed significant differences (*p*-value < 0.05) between all MEL-treated fruits and the control, with values of 18.99 ± 0.57% for the control and 10.24 ± 0.26% in lemons treated with 1 mM MEL. Comparing LEAF and NO LEAF lemons, there were significant differences (*p*-value < 0.05), with an exception for the control after storage at Harvest 1. Nevertheless, there were no significant differences at Harvest 2.

Chilling injury is a physiological disorder that manifests itself in fruit after it is exposed to temperatures close to the freezing point. Cold tolerance depends on several factors such as the species, cultivar, harvest time, temperature and time of exposure to cold storage [48,49]. In a study in which the crown plays an important role in maintaining the quality of the harvested pineapple, it was appreciated that the crown offered greater protection from cold damage [50]. This may be due to the higher contribution of metabolic compounds from the pineapple leaf. MEL treatments produced significant reductions in electrolyte leakage, showing a dose-dependent effect. Other studies have shown that melatonin is able to reduce lipid peroxidation of membranes by improving cell integrity and the ability in peaches and cabbage to maintain a balance in the cells of oxidative metabolism [51,52].

### 3.3. Effect of Melatonin on Total Phenolic

**Figure 7:**
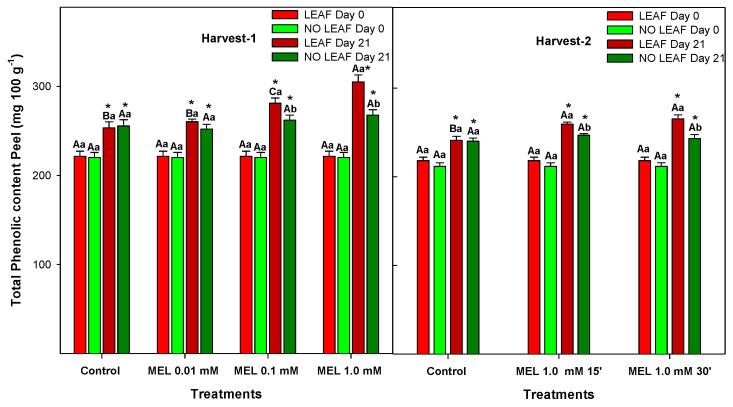
Total phenolic content in peels of ‘Fino’ lemons with LEAF and NO LEAF as affected by MEL treatments at 0.01, 0.1 and 1.0 mM (Harvest 1) and MEL at 1.0 mM during 15 and 30 min (Harvest 2) after 21 days of storage. Data are the mean ± SE. Bars with different capital letters denote significant differences between control and MEL treatments, while bars with different small letters denote significant differences between lemons with LEAF and NO LEAF. The asterisk symbol denotes significant differences between both storage days (0 and 21 days) for each type of leaf and treatment.

**Figure 8:**
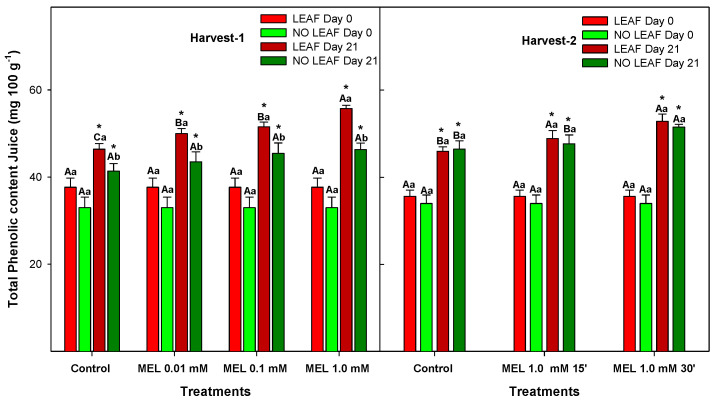
Total phenolic content in juice of ‘Fino’ lemons with LEAF and NO LEAF as affected by MEL treatments at 0.01, 0.1 and 1.0 mM (Harvest 1) and MEL at 1.0 mM during 15 and 30 min (Harvest 2) after 21 days of storage. Data are the mean ± SE. Bars with different capital letters denote significant differences between control and MEL treatments, while bars with different small letters denote significant differences between lemons with LEAF and NO LEAF. The asterisk symbol denotes significant differences between both storage days (0 and 21 days) for each type of leaf and treatment.

Citrus fruits are known to contain a wide variety of phytochemical compounds with benefits for human health demonstrated by clinical and epidemiological studies, such as reduction of cardiovascular diseases, cancers and diabetes, among others [53]. In lemons, total phenolic content was analysed in both the peel (Figure 7) and the juice (Figure 8), and concentration was significantly higher (10 fold) in the peel than in the juice (*p*-value < 0.05), although they share the same behaviour, which is an increase during 21 days of storage; levels were significantly higher (*p*-value < 0.05) in LEAF that in NO LEAF fruits, and MEL treatment induced greater accumulation of total phenolics.

In lemons, the main phytochemicals are flavonoids and are found at high concentration in both peel and pulp [54]. The biological activities of phenolic compounds are well documented as acting as antioxidant moieties. The role of MEL modulating the content of total phenolics has been documented in citrus and other fruits. Thus, in oranges, MEL also showed an accumulation of total phenolics during storage, leading to maintenance of postharvest quality and in turn alleviating physiological senescence [20]. In mandarins, MEL at 250, 500 and 1000 μM showed that the 1000 μM treatment was more effective than other doses during storage on maintaining higher total antioxidant activity as well as total phenolics [36]. In the same way, mangoes treated with MEL at 10, 100 or 1000 μM showed significantly higher total phenol content for MEL at 1000 μM than the control, while 10 or 100 μM did not significantly influence total phenol content [55], which agrees with our results in ‘Fino’ lemons, where MEL at 1.0 mM was the best concentration for increasing total phenolics.

## 4. Conclusions

The postharvest treatments with melatonin by immersion in ‘Fino’ lemons had a positive effect on the improvement of the quality and in the late ripening of the lemon, reducing weight loss or the rate of breathing of the fruit as well as increasing firmness or colour levels and decreasing electrolyte leakage. The most effective dose was MEL 1 mM, which showed better results. In addition, LEAF lemons showed in general that they could maintain better quality attributes with respect to NO LEAF lemons, possibly due to a greater absorption of melatonin through the leaf. For the content of phenolic compounds in both skin and juice, we observed the same positive effect on both parameters (treatment and type of leaf). However, further research including the effect of the combination of these treatments with different times of immersion and the effect of MEL application by immersion in LEAF and NO LEAF fruits in order to elucidate the way of introduction inside them is needed.

## Data Availability

The data presented in this study are available on request from the corresponding author (daniel.valero@umh.es/fbadiche@umh.es). The data are not publicly available because we are still pursuing this line of study, and their publication must be authorised by the Ministry of Science and Innovation.

## Supplementary Materials

The following supporting information can be downloaded at https://www.mdpi.com/article/10.3390/foods12152979/s1: Figure S1: Total soluble solids of ‘Fino’ lemons with LEAF and NO LEAF as affected by MEL treatments at 0.01, 0.1 and 1.0 mM (Harvest 1) and MEL at 1.0 mM during 15 and 30 min (Harvest 2) after 21 days of storage. Data are the mean ± SE. Bars with different capital letters denote significant differences between control and MEL treatments, while bars with different small letters denote significant differences between lemons with LEAF and NO LEAF; Figure S2: Titratable acidity of ‘Fino’ lemon with LEAF and NO LEAF as affected by MEL treatments at 0.01, 0.1 and 1.0 mM (Harvest-1) and MEL at 1.0 mM during 15 and 30 minutes (Harvest-2) after 21 days of storage. Data are the mean ± SE. Bars with different capital letter denote significant differences between control and MEL treatments, while bars with different small letter denote significant differences between lemons with LEAF and NO LEAF. Asterisk symbol denotes significant differences between both storage days (0 and 21 days) for each type of leaf and treatment.
