# Supplementary material for: Melatonin Postharvest Treatment in Leafy ‘Fino’ Lemon Maintains Quality and Bioactive Compounds
Source: Foods. 2023 Aug 7;12(15):2979. doi: 10.3390/foods12152979 (PMC10418853; doi:10.3390/foods12152979)
Supplement: Supplementary file 1 [file foods-12-02979-s001.zip › foods-2526734-supplementary.pdf]

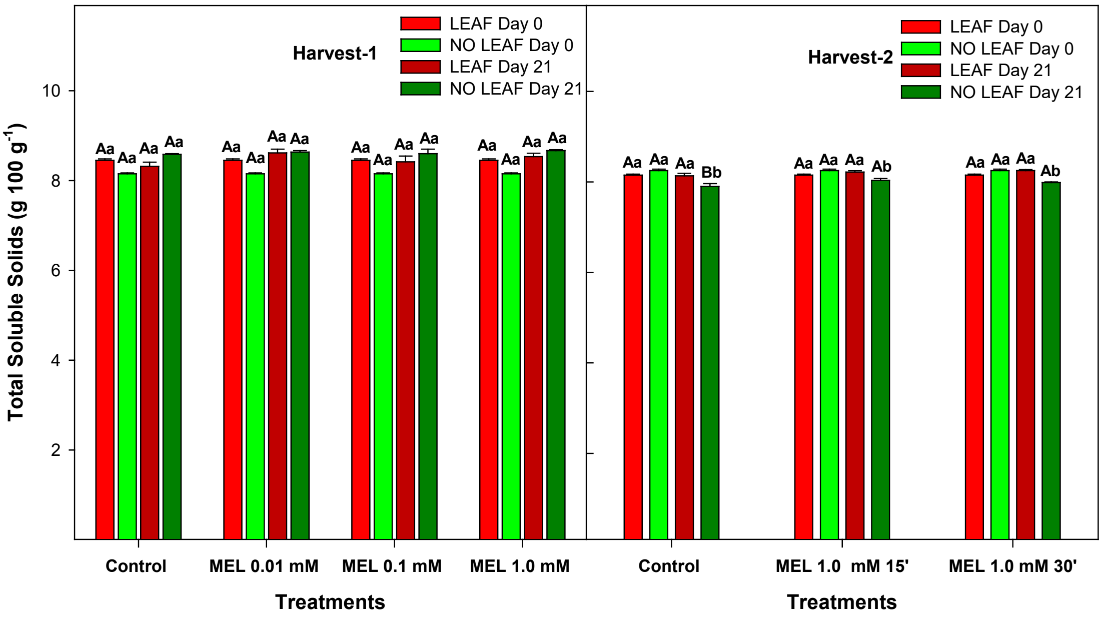

Figure S1. Total soluble solids of 'Fino' lemon with LEAF and NO LEAF as affected by MEL treatments at 0.01, 0.1 and 1.0 mM (Harvest-1) and MEL at 1.0 mM during 15 and 30 minutes (Harvest-2) after 21 days of storage. Data are the mean ± SE. Bars with different capital letter denote significant differences between control and MEL treatments, while bars with different small letter denote significant differences between lemons with LEAF and NO LEAF. Asterisk symbol denotes significant differences between both storage days (0 and 21 days) for each type of leaf and treatment.

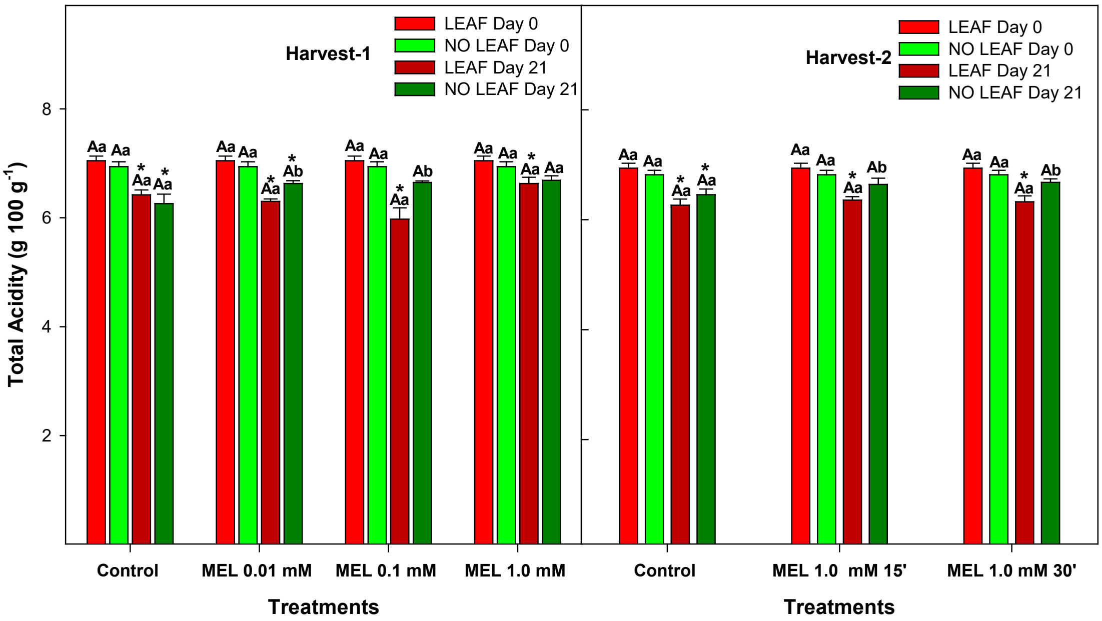

Figure S2. Titratable acidity of 'Fino' lemon with LEAF and NO LEAF as affected by MEL treatments at 0.01, 0.1 and 1.0 mM (Harvest-1) and MEL at 1.0 mM during 15 and 30 minutes (Harvest-2) after 21 days of storage. Data are the mean ± SE. Bars with different capital letter

denote significant differences between control and MEL treatments, while bars with different small letter denote significant differences between lemons with LEAF and NO LEAF. Asterisk symbol denotes significant differences between both storage days (0 and 21 days) for each type of leaf and treatment.
